# IL-6 deletion decreased REV-ERBα protein and influenced autophagy and mitochondrial markers in the skeletal muscle after acute exercise

**DOI:** 10.3389/fimmu.2022.953272

**Published:** 2022-10-13

**Authors:** Ana P. Pinto, Vitor R. Muñoz, Alisson L. da Rocha, Rafael L. Rovina, Gustavo D. Ferrari, Luciane C. Alberici, Fernando M. Simabuco, Giovana R. Teixeira, José R. Pauli, Leandro P. de Moura, Dennys E. Cintra, Eduardo R. Ropelle, Ellen C. Freitas, Donato A. Rivas, Adelino S. R. da Silva

**Affiliations:** ^1^ School of Physical Education and Sport of Ribeirão Preto, University of São Paulo (USP), São Paulo, Brazil; ^2^ Postgraduate Program in Rehabilitation and Functional Performance, Ribeirão Preto Medical School, University of São Paulo (USP), São Paulo, Brazil; ^3^ Department of Biomolecular Sciences, School of Pharmaceutical Sciences of Ribeirao Preto, Faculty of Pharmaceutical Sciences of Ribeirao Preto, University of São Paulo (FCFRP USP), Sao Paulo, Brazil; ^4^ Laboratory of Molecular Biology of Exercise (LaBMEx), School of Applied Sciences, University of Campinas (UNICAMP), São Paulo, Brazil; ^5^ Department of Biochemistry, Federal University of São Paulo (UNIFESP), São Paulo, Brazil; ^6^ Multicentric Program of Postgraduate in Physiological Sciences, School of Dentistry of Araçatuba, São Paulo State University (UNESP), São Paulo, Brazil; ^7^ Department of Physical Education, State University of São Paulo (UNESP), São Paulo, Brazil

**Keywords:** genetic deletion, Nr1d1, autophagic flux, mitochondria, C2C12 cells, pharmacological treatment

## Abstract

Interleukin 6 (IL-6) acts as a pro and anti-inflammatory cytokine, has an intense correlation with exercise intensity, and activates various pathways such as autophagy and mitochondrial unfolded protein response. Also, IL-6 is interconnected to circadian clock-related inflammation and can be suppressed by the nuclear receptor subfamily 1, group D, member 1 (*Nr1d1*, protein product REV-ERBα). Since IL-6 is linked to physical exercise-modulated metabolic pathways such as autophagy and mitochondrial metabolism, we investigated the relationship of IL-6 with REV-ERBα in the adaptations of these molecular pathways in response to acute intense physical exercise in skeletal muscle. The present study was divided into three experiments. In the first one, wild-type (WT) and IL-6 knockout (IL-6 KO) mice were divided into three groups: Basal time (Basal; sacrificed before the acute exercise), 1 hour (1hr post-Ex; sacrificed 1 hour after the acute exercise), and 3 hours (3hr post-Ex; sacrificed 3 hours after the acute exercise). In the second experiment, C2C12 cells received IL-6 physiological concentrations or REV-ERBα agonist, SR9009. In the last experiment, WT mice received SR9009 injections. After the protocols, the gastrocnemius muscle or the cells were collected for reverse transcription-quantitative polymerase chain reaction (RTq-PCR) and immunoblotting techniques. In summary, the downregulation of REV-ERBα, autophagic flux, and most mitochondrial genes was verified in the IL-6 KO mice independent of exercise. The WT and IL-6 KO treated with SR9009 showed an upregulation of autophagic genes. C2C12 cells receiving IL-6 did not modulate the *Nr1d1* mRNA levels but upregulated the expression of some mitochondrial genes. However, when treated with SR9009, IL-6 and mitochondrial gene expression were upregulated in C2C12 cells. The autophagic flux in C2C12 suggest the participation of REV-ERBα protein in the IL-6-induced autophagy. In conclusion, the present study verified that the adaptations required through physical exercise (increases in mitochondrial content and improvement of autophagy machinery) might be intermediated by an interaction between IL-6 and REVERBα.

## Introduction

Interleukin 6 (IL-6) is a pleiotropic cytokine with roles in acute-phase response, skeletal mass, and metabolism (1). IL-6 binding to IL-6 receptors (IL-6R) and generates the activation of transmembrane glycoprotein 130 (gp130). The gp130 can activate a variety of pathways, such as phosphatidylinositol 3-kinase (PI3K), AMP-activated protein kinase (AMPK), and signal transducer and activator of transcription 3 (STAT3) (2). IL-6 was initially characterized as a proinflammatory cytokine with roles in immune responses, inflammation, hematopoiesis, and oncogenesis, regulating cell growth, survival, and differentiation (3, 4). The current consensus is that IL-6 from skeletal muscle has anti-inflammatory and regenerative functions, especially when released during exercise. Exercise duration, intensity, and exercise-induced muscle damage can influence IL-6 release (5). In response to physical activity, IL-6 works as a vital ligand of muscle contraction and regulates hepatic glucose output (6). However, in response to exhaustive exercise, the increase of IL-6 in the serum is associated with an inflammatory response (7).

Moderate endurance exercise activates AMPK due to an increased AMP/ATP ratio (8). AMPK phosphorylates and activates peroxisome proliferator-activated receptor-γ coactivator 1α (PGC1α), increasing mitochondrial content, skeletal muscle oxidative capacity, and maximal oxygen uptake (VO_2_max) (8, 9). Proper mitochondrial functioning is dependent on the mitochondrial unfolded protein response (UPRmt) (10). Woldt et al. demonstrated that the absence of the nuclear receptor subfamily 1, group D, member 1 (*Nr1d1*, protein product REV-ERBα) decreases the mitochondrial content and oxidative function, compromising exercise capacity, but upregulates autophagy (11). The REV-ERBα is a transcriptional silencer that suppresses the expression of genes involved in several physiological roles, such as inflammation, metabolism, and circadian rhythm (12).

Gibbs et al. (13) demonstrated that administering a REV-ERBα ligand or the genetic knockdown of REV-ERBα expression in macrophage is efficient at regulating the production and release of IL-6. However, the ability of IL-6 to interfere in the production and release of REV-ERBα in skeletal muscle is unclear and needs more understanding. In macrophages, REV-ERBα acts as a repressor of the inflammatory response inhibiting IL-6 (14). IL-6 is interconnected with circadian clock-related inflammation (15). Daily patterns of immune cell functions, including the timing of leukocyte trafficking and inflammatory signaling, are tuned by the circadian clock. This regulation helps coordinate the effectiveness of inflammatory response through exposure to pathogens and inflammatory insults (15).

Since REV-ERBα inhibits IL-6, the first objective of this study was to verify the effects of the global deletion of IL-6 on the mRNA level and protein content of REV-ERBα in the skeletal muscle of these transgenic mice. Knowing that aerobic exercise can modulate IL-6 and REV-ERBα (9, 11), which are related to autophagy and UPRmt pathways (11, 16), our second objective was to verify if the effects on REV-ERBα are dependent on IL-6 and how those skeletal muscle molecular pathways are affected. We also treated C2C12 cells with IL-6 or SR9009 (an agonist of REV-ERBα) to evaluate the responses of critical genes of autophagy and mitochondrial pathways. Finally, those essential genes were measured in gastrocnemius samples of WT mice receiving 3 days of SR9009 injections.

## Materials and methods

### Experimental animals

**Figure 1:**
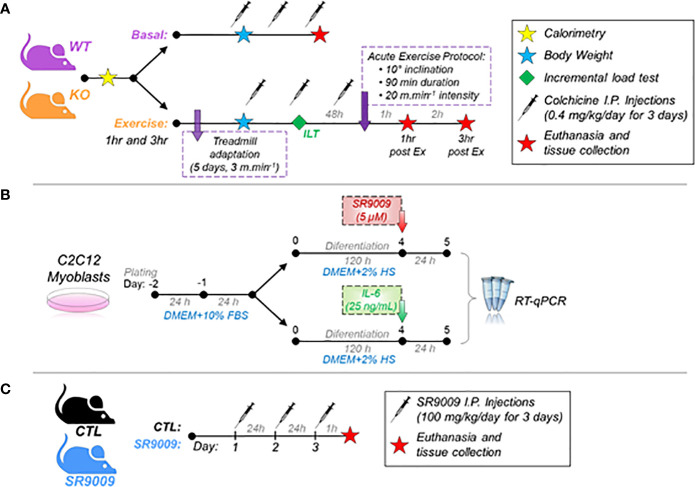
A schematic diagram of the experiments. **(A)** Experimental design of WT and IL-6 KO mice at basal and 1hr and 3hr post-exercise. A different batch of mice received colchicine I.P injections; **(B)** Experimental design of C2C12 myoblasts treated with SR9009 or IL-6; **(C)** Experimental design of CTL and SR9009 administration in mice. WT, wild-type mice; IL-6 KO, interleukin 6 knockout mice; CTL, control group.

Eight-week-old C57BL/6 male mice from the University of São Paulo (USP) at the Central Animal Facility of the Ribeirão Preto campus were used for the wild-type (WT) group. For the IL-6 knockout (KO) group, eight-week-old IL-6^-/-^ male mice from the Laboratory of Molecular Immunology and Embryology, Transgenose Institute, *Centre National de la Recherche Scientifique* (CNRS) were bred onto a C57BL/6 background and used for the other experimental group. The male gender was used to avoid the influence of the hormonal cycle of female mice since the responses of the circadian cycle can suffer interference (17). The Ethics Committee of the University of Sao Paulo (I.D. 2018.5.70.90.0) approved all the experimental procedures, which were performed according to the Brazilian College of Animal Experimentation (COBEA). The animals were kept in sterile micro-insulators (two to three animals per cage) in a ventilated rack (INSIGHT™, Ribeirão Preto, SP, Brazil) with a controlled temperature (22 ± 2°C) on a 12:12-h light-dark inverted cycle (lights on at 6 pm and lights off at 6 am) with water and food (Purina chow) provided *ad libitum*. The WT and KO mice were divided into three groups: Basal time (Basal, n=5; sacrificed before the acute exercise), 1 hour (1hr post-Ex, n=5; sacrificed 1 hour after the acute exercise), and 3 hours (3hr post-Ex, n=5; sacrificed 3 hours after the acute exercise). **Figure 1A** shows the experimental design.

### Indirect calorimetry

Five mice of each group (i.e., WT and IL-6 KO) were submitted to the indirect calorimetry before any procedure. The exam was executed using the Panlab Oxylet System (Panlab, S.L., Barcelona, Spain). For 48 h, the rodents were accommodated in acrylic cages (three animals per cage) at a controlled temperature (22 ± 2° C). The mice were adapted to the apparatus in the first 24 h. In the other 24 h, the following variables were measured: oxygen consumption (VO_2_), carbon dioxide production (VCO_2_), total energy expenditure (EE), and respiratory quotient (RQ), which was determined by the VCO_2_/VO_2_ ratio.

### Incremental load test

The exercised groups were adapted to the treadmill running (INSIGHT^®^, Ribeirão Preto, São Paulo, Brazil) for 10 min.day^-1^ at 3 m.min^-1^ for five days. After one week of adaptation, the ILT test was performed. The treadmill was set up at 6 m.min^-1^ at 10 degrees of inclination with increments of 3 m.min^-1^ every 3 min until exhaustion, which was established when mice touched the end of the treadmill five times in 1min. The ILT was used to compare the aerobic capacity of the WT and IL-6 KO groups.

### Acute exhaustive physical exercise protocol

After 48 h of the ILT test, the exercised mice (WT and IL-6 KO groups) performed an acute exhaustive physical exercise protocol on the treadmill running with an intensity of 20 m.min^-1^ at 10 degrees of inclination for 90 min (18). This acute exhaustive physical exercise protocol was selected because IL-6 serum levels were higher after 1 and 3 hours than baseline (19).

### Gastrocnemius extraction

Rodents were weighed using an analytical balance (Toledo, São Bernardo do Campo, São Paulo, Brazil). After that, mice were anesthetized with an intraperitoneal injection of xylazine (10 mg/kg of body weight) and ketamine (100 mg/kg of body weight) before the acute exercise, at 1 hour and 3 hours after the acute exercise. As soon as the loss of pedal reflexes confirmed the effect of anesthesia, gastrocnemius muscle was removed and prepared for reverse transcription-quantitative polymerase chain reaction (RTq-PCR; n = 5 mice for each experimental group) and immunoblotting technique (n = 5 mice for each experimental group).

### Autophagic flux

A different batch of mice was divided into two groups: Basal (WT and KO mice) and Trained (WT and KO mice submitted to the acute exhaustive physical exercise protocol) with intraperitoneal injections of vehicle (0.9% saline) or colchicine (0.4 mg/kg/day: AB120663, ABCAM, Cambridge, UK) for 3 consecutive days. The final injection was performed 1h before the acute physical exercise protocol (20–23). After the exercise protocol and totalizing three hours from the last injection, the animals were anesthetized by an intraperitoneal administration of xylazine (10 mg/kg body weight) and ketamine (100 mg/kg body weight). As soon as the loss of pedal reflexes confirmed the effect of anesthesia, the gastrocnemius was removed, washed with saline, and used for the immunoblotting technique. The autophagic flux index was calculated as follows: Autophagy flux index = (LC3II expression levels with colchicine)/(LC3II expression levels without colchicine) (24). Each LC3II was normalized by its beta-actin expression level (24).

### Reverse transcription-quantitative polymerase chain reaction

Gastrocnemius samples were collected and stored in RNAlater solution (Ambion, Foster City, CA). All procedures were performed under standard RNase-free conditions to avoid exogenous RNase contamination. Total RNA was extracted using TRIzol^®^ Reagent (Thermo Fischer Scientific, Waltham, MA) according to the manufacturer´s instructions. Total RNA was quantified by spectrophotometer at OD 260, and quality was checked by the OD 260/280 ratio (BioDrop µLite, Biochrom, Holliston, MA). cDNA was synthesized using a High-Capacity cDNA Reverse Transcription Kit (Applied Biosystems, Foster City, CA).

Quantitative real-time PCR was performed using StepOne Plus PCR System (Applied Biosystems) to analyze the relative mRNA expression of Nuclear Receptor Subfamily 1 Group D Member 1 (*Nr1d1*), Protein Kinase AMP-Activated Catalytic Subunit Alpha 1 (*Prkaa1*), Peroxisome Proliferator-Activated Receptor Gamma Coactivator 1-Alpha (*Ppargc1-alpha*), Unc-51 like autophagy activating kinase 1 (*Ulk1*), Beclin 1 (*Becn1*), Autophagy Related 5 (*Atg5*), Microtubule Associated Protein 1 Light Chain 3 Beta (*Map1lc3*), Sequestosome 1 (*Sqstm1*), BCL2 Interacting Protein 3 (*Bnip3*), Transcription Factor A, Mitochondrial (*Tfam*), Nuclear respiratory factor 1 (*Nrf1*), Mitochondrially Encoded Cytochrome C Oxidase I (*Mtco1*), Nuclear Receptor ROR-Alpha (*Rorα*), cAMP-dependent Protein Kinase Catalytic Subunit Alpha (*Pka*), Signal Transducer And Activator Of Transcription 6 (*Stat6*), Peroxisomal Acyl-Coenzyme A oxidase 1 (*Acox*), Carnitine O-palmitoyltransferase 1 (*Cpt1*) (**Table 1**).

**Table 1:** Primers sequences used in this study.

Gene	Forward (5’-3’)	Reverse (5’-3’)
*Nr1d1*	GGTGTTTGGCGCAGCACCTT	CTCTGGGATAAATGCCCGAAGCT
*Prkaa1*	CCAGGTCATCAGTACACCATCT	TTTCCTTTTCGTCCAACCTTCC
*Ppargc1-alpha*	GAGTTGAAAAAGCTTGACTGGC	CAGCACACTCTATGTCACTCCA
*Ulk1*	AACATCCGAGTCAAGATTGCTG	ATAATGACCTCAGGAGCCATGT
*Becn1*	AGGAACTCACAGCTCCATTACT	CTCTCCTGAGTTAGCCTCTTCC
*Atg5*	GCTTTTGCCAAGAGTCAGCTAT	AACCAATTGGATAATGCCATTTCAG
*Map1lc3*	AGATAATCAGACGGCGCTTG	TCGTACACTTCGGAGATGGG
*Sqstm1*	ACAGCCAGAGGAACAGATGG	GTAGAGACTGGAGTTCACCTGTA
*Bnip3*	CAGCATGAGAAACACAAGCG	TCCAATGTAGATCCCCAAGCC
*Tfam*	GCAAAGGATGATTCGGCTCAGGGAA	CCGGATCGTTTCACACTTCGACGG
*Nrf1*	GGTGTTTGGCGCAGCACCTT	CTCTGGGATAAATGCCCGAAGCT
*Mtco1*	ACTATACTACTAACAGACCG	GGTTCTTTTTTTCCGGAGTA
*Rorα*	GATCCTGGACAATACCTCGGAG	GACATCCGACCAAACTTGACA
*Pka*	CGGCGGATTGGAAGGTTCA	CCTGCTGGTCGATGAGAAGATT
*Stat6*	CCTGCTGGTCGATGAGAAGATT	CATCTGAACCGACCAGGAACT
*Acox*	AGGTGAAAGCCTTCAGTCCAGC	AGGTGAAAGCCTTCAGTCCAGC
*Cpt1*	CTCCACAGCATCAAGAGACTGC	GATCCTGGACAATACCTCGGAG
*Gapdh*	AAGAGGGATGCTGCCCTTAC	CGGGACGAGGAAACACTCTC

The amplification reactions (10 µL final volume) were performed in duplicate with the following reagents: 5 µL 2× Power Sybr Master Mix (Thermo Fisher Scientific, Wilmington, DE, USA), 1uL (1 uM) primer forward, 1 µL (1 uM) primer reverse, 1 µL cDNA diluted in 1:10 and 2 µL of DPEC water. *Gapdh* was used as a reference gene for the normalization of the data. Each amplification reaction occurred in the standard cycling in the following cycles: 10 minutes at 95° C and a further 40 cycles with 15 seconds at 95° C and 1 minute at 60° C. Relative quantification was calculated by the 2 -ΔΔCT method using the Thermo Fisher Cloud Software, RQ version 3.7 (Life Technologies Corporation, Carlsbad, CA, USA).

### Immunoblotting technique

The immunoblotting technique was performed as previously described by our research group (25–28). The antibodies used were: Glyceraldehyde-3-phosphate dehydrogenase (GAPDH; #2118), Microtubule Associated Protein 1 Light Chain 3 Beta (LC3; #3868), Signal Transducer And Activator Of Transcription 3 (STAT3; #30835), Phospho Signal Transducer And Activator Of Transcription 3 (p-STAT3; #4113) and AMP-activated protein kinase (AMPK; #2532) from Cell Signaling Technology (Cell Signaling Technology, MA, USA); Nuclear Receptor Subfamily 1 Group D Member 1 (REV-ERBα; SC-393215), Heat Shock protein 60 (Hsp60; SC-13115) and Phospho AMP-activated protein kinase (p-AMPK; SC-33524) from Santa Cruz Biotechnology (Dallas, Tx, USA); Nuclear Respiratory Factor (Nrf1; ab-55774), Transcription Factor A, Mitochondrial (TFAM; ab-131607), Mitochondrially encoded NADH: ubiquinone oxidoreductase core subunit 1 (Mt-ND1; ab-181848) and Oxphos (Oxphos; ab-110413) from ABCAM (Cambridge, UK). The primary antibodies were utilized at a dilution of 1:1,000, and the secondary antibodies were used at a dilution between 1:10,000 and 1:20,000. Images were acquired by the C-Digit Blot Scanner (LI-COR, Lincoln, Nebraska, USA) and quantified using the software Image Studio for C-DiGit™ Blot Scanner. After the band intensities were measured, all proteins (phosphorylated or not) were normalized by a control protein (GAPDH). Then the phosphorylated/non-phosphorylated ratio was calculated. A Ponceau S-stain was used as an experiment loading control. Figure and supplementary figure panels were checked for data integrity using the Proofig pipeline (https://www.proofig.com).

### C2C12 cells treatment

The C2C12 myoblasts (ATCC^®^ CRL-1772™) were grown in Dulbecco’s Modified Eagle’s Medium (DMEM, #12100046, ThermoFisher scientific^®^) with 10% of fetal bovine serum (FBS - Gibco^®^ #A476680), 1% of Penicillin/Streptomycin complexes (P/S – Gibco^®^ #15140122) at 37˚ C, 5% CO_2_. The cells were seeded (3 _x_ 10^4^ cells/well) in a 24-well plate, and after reaching 90% of confluence, the differentiation into myotubes was initiated using DMEM supplemented with 2% horse serum for 5 days. On the 5th day after differentiation, the cells were treated with recombinant mouse IL-6 (25 ng/mL, #50136-MNAE from Sino Biological Inc^®^) or SR9009 (5 µM, #11929 from Cayman^®^) for 24 h. Koliphor (#C5135 from Sigma^®^) was used as vehicle control in the SR9009 treatment. After treatment, the cells were collected with 400 µL Trizol (Life Technologies^®^, USA) and submitted to RNA extraction protocol for the RT-q-PCR. **Figure 1B** shows the experimental design.

### Autophagic flux using ptfLC3 plasmid

To measure the autophagosome vesicles and autophagic flux, C2C12 myoblasts were seeded (5x10^4^ cells/well) in a 12-well plate. In one experiment, the cells were transfected with negative control siRNA (siNT) or Rev-Erbα siRNA (15 pmol/well) with Lipofectamine RNAiMAX (#13778, Invitrogen^®^). After 24h, the cells were transfected with ptfLC3 plasmid (1 µg/well, #21074, Addgene^®^) using Lipofectamine 3000 (#L3000001, Invitrogen^®^). Six hours after the plasmid transfection, the cells were treated with recombinant mouse IL-6 (25 ng/mL, #50136-MNAE from Sino Biological Inc^®^) for another 24 periods and the images were acquired in a Motic™ AE31E Trinocular Microscope attached with Motic MXH-100 power supply and Moticam ProS5 Plus and Motic Images Plus 3.0 software (at 40x magnification).

In another experiment, C2C12 myoblasts seeded in a 12-well plate were transfected with ptfLC3 plasmid. After six hours, the cells were treated for 24h with vehicle or SR9009 (5 µM) in combination or not with pharmacological IL-6 receptor neutralizing antibody (Tocilizumab, 10 µg/mL). Finally, the images were acquired as described before.

### SR9009 administration in mice

Six C57BL/6 male mice and five IL-6^-/-^ mice at 10-week-old were used for treatment with SR9009 (DC9544 – DC Chemicals, Shanghai, China) (100 mg/kg, i.p.) diluted in Kolliphor EL 15% (C5135, Sigma). Six C57BL/6 male mice and five IL-6^-/-^ mice were used as control grouped and treated with the vehicle solution (Kolliphor EL 15%). Injections were performed twice daily (6:00 am and 12:00 pm; ZT12 and ZT18, respectively) for 3 consecutive days (16). These times were chosen due to the higher expression of *Nr1d1* during the circadian cycle. The animals received the last dose at noon on the last day of treatment. After 1 hour, the mice were anesthetized with an intraperitoneal injection of xylazine (10 mg/kg of body weight) and ketamine (100 mg/kg of body weight). As soon as the loss of pedal reflexes confirmed the effect of anesthesia, the gastrocnemius muscle was removed and prepared for the RTq-PCR. **Figure 1C** shows the experimental design.

### Statistical analysis

Results are expressed as mean ± standard error of the mean (SEM). Levene’s test was used to verify the homogeneity of variances, and the Shapiro–Wilk *W*-test was used to check data normality. The western blotting results were submitted to the identifiers outliers by the Grubbs’ method (alpha=0.05). When normality was confirmed, the two-way analysis of variance (ANOVA) was used to compare a specific gene/protein expression response between exercise time and groups for the first experimental design (**Figure 1A**). Bonferroni’s *post hoc* test was performed when the two-way ANOVA indicated significance. For results from experimental designs two and three, an unpaired Student’s *t*-test was applied to investigate the possible differences between the experimental groups. All statistical analyses were set at p ≤ 0.05 and two-sided. Statistical analyses were performed using GraphPad Prism v.8.0.1 for Windows (GraphPad Software, CA, USA).

## Results

### The IL-6 KO group showed higher calorimetry measures.

**Figure 2:**
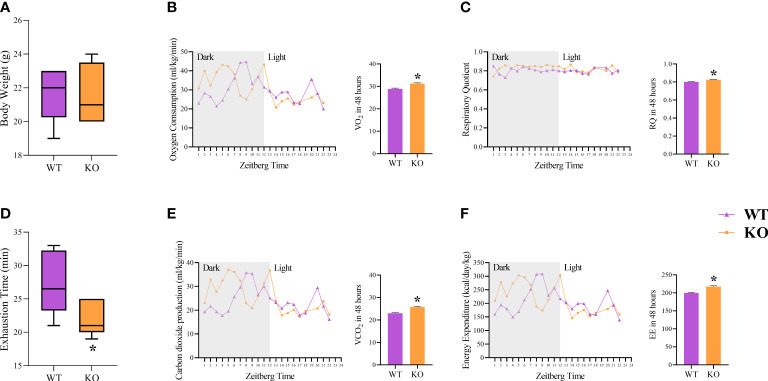
Metabolic outcomes and calorimetry. Body weight (g) **(A)**; Exhaustion time (min) **(B)**; Oxygen Consumption (ml/kg/min) (VO_2_) **(C)**; Carbon dioxide production (ml/kg/min) (VCO_2_) **(D)**; Respiratory Quotient (RQ) **(E)**; Energy Expenditure (kcal/day/kg) (EE) **(F)**. Data correspond to the mean ± SEM of n=5 mice/group. *p ≤ 0.05 *vs.* WT. WT (sedentary; Basal), IL-6 KO (sedentary; Basal). The line graphs correspond to 24 hours, and the bar graphs correspond to 48 hours.

The body weight was not different between the WT and KO groups (**Figure 2A**). The incremental load test was lower for the IL-6 KO group (**Figure 2B**). The IL-6 KO group showed higher values of all the variables measured (**Figures 2C–F**): oxygen consumption (VO_2_), carbon dioxide production (VCO_2_), total energy expenditure (EE), and respiratory quotient (RQ: mean: 0.800 for WT and 0.825 for the IL-6 KO).

### The IL-6 KO mice showed upregulation of Nr1d1, downregulation of REV-ERBα at basal condition, and downregulation of autophagic flux index.

**Figure 3:**
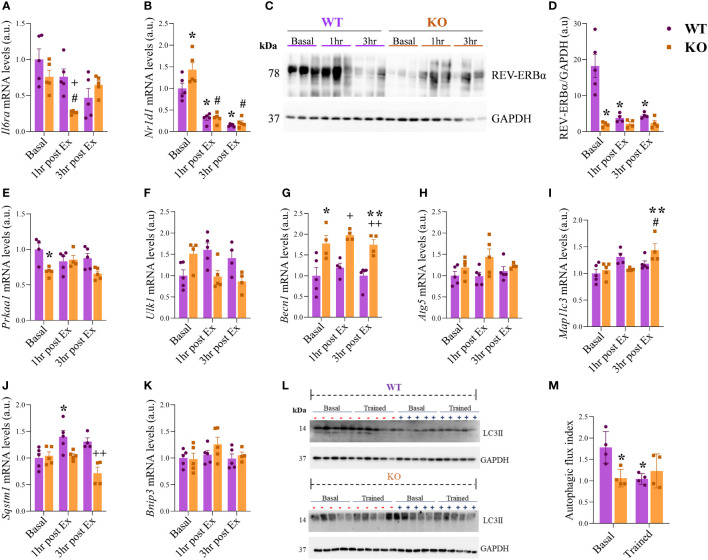
mRNA and protein levels of *Nr1d1* and autophagic pathway. *Il6ra* **(A)**; *Nr1d1* **(B)**; Representative blots **(C)**; REV-ERBα/GAPDH **(D)**; *Prkaa1* **(E)**; *Ulk1* **(F)**; *Becn1* **(G)**; *Atg5* **(H)**; *Mapllc3* **(I)**; *Sqstm1* **(J)**; *Bnip3* **(K)**; Representative membranes of the autophagic flux **(L)**; Autophagic flux index **(M)**. Data correspond to the mean ± SEM of n=4-5 mice/group for RT-qPCR and n=5-6 mice/group for western blotting. *p ≤ 0.05 *vs.* WT at Basal; #p ≤ 0.05 *vs.* IL-6 KO at Basal; +p≤ *vs.* WT at 1hr post-Ex; **p ≤ 0.05 *vs.* KO at 1hr post-Ex; ++p≤ *vs.* WT at 3hr post-Ex. Basal (sedentary; Basal), 1 hour (after 1h of the acute exercise; 1hr post-Ex), and 3 hours (after 3h of the acute exercise; 3hr post-Ex). a.u., arbitrary units.

The *Il6ra* mRNA levels were lower for the IL-6 KO group at 1hr post-Ex when compared to WT at 1hr post-Ex and KO at the baseline (**Figure 3A**). The *Nr1d1* mRNA levels (**Figure 3B**) were higher for the IL-6 KO group at basal when compared to WT at basal and KO at 1hr and 3hr post-Ex. Also, the *Nr1d1* mRNA levels were downregulated after exercise (1hr and 3hr post-Ex) for the WT group compared to WT basal condition. **Figure 3C** shows the representative membranes of REV-ERBa and GAPDH. The total REV-ERBα protein levels (**Figure 3D**) were lower for the IL-6 KO group at the basal when compared to WT under the same situation. The WT 1hr and 3hr post-Ex showed lower levels than WT at basal condition. The *Prkaa1* mRNA levels (**Figure 3E**) were downregulated for the KO group at basal compared to WT at the same time. The *Ulk1*, *Atg5*, and *Bnip* mRNA levels were not different between the groups and the times (**Figures 3F, H, K**). The *Becn1* mRNA levels (**Figure 3G**) were higher for the KO group at Basal, 1hr post-Ex, and 3hr post-Ex compared to the WT group at the same time. Also, the *Becn1* mRNA levels were lower for the KO group at 3hr post-Ex when compared to 1hr post-Ex. The *Mapllc3* mRNA levels (**Figure 3I**) were higher for the KO group at 3hr post-Ex when compared to Basal and 1hr post-Ex. The *Sqstm1* mRNA levels (**Figure 3J**) were higher for the WT group at 1hr post-Ex when compared to Basal. Also, the KO at 3hr post-Ex had lower levels when compared to the WT group at the same time. For the autophagic flux index (representative membranes in **Figure 3L**), the LC3II protein levels were lower for the KO at basal and WT trained when compared to WT at basal (**Figure 3M**). The *Rorα*, *Pka*, *Stat6*, and *Acox* mRNA levels were not different among the groups and the times (**Supplementary File Figures 6A–D**). The *Cpt1* mRNA levels were higher for the WT group at 1hr post-Ex and lower for the WT group at 3hr post-Ex group when compared to WT at baseline. Also, the IL-6 KO group at 1hr post-Ex and WT group at 3hr post-Ex had lower levels when compared to the WT group at 1hr post-Ex. IL-6 KO group at 1hr post-Ex had lower levels when compared to the IL-6 KO group at baseline (**Supplementary File Figure 6E**). The protein levels of p-STAT3/STAT3, p-AMPK/AMPK and ATG12 were not different between the groups and the times (**Supplementary File Figures 7B, 8B**).

### Genes involved in mitochondrial metabolism were downregulated for the IL-6 KO group, independent of exercise.

**Figure 4:**
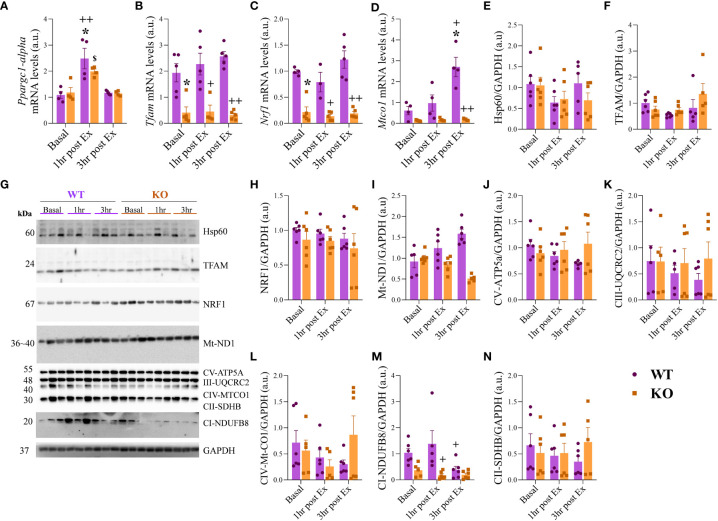
mRNA and protein levels related to mitochondrial metabolism. *Ppargc1-alpha* **(A)**; *Tfam* **(B)**; *Nrf1* **(C)**; *Mtco1* **(D)**. Hsp60/GAPDH **(E)**; TFAM/GAPDH **(F)**; Representative membranes **(G)**; NRF1/GAPDH **(H)**; Mt-ND1/GAPDH **(I)**; CV-ATP5a/GAPDH **(J)**; CII-UQCRC2/GAPDH **(K)**; CIV-Mt-CO1/GAPDH **(L)**; CII-SDHB/GAPDH **(M)**; CI-NDUFB8/GAPDH **(N)**. Data correspond to the mean ± SEM of n=4-5 mice/group for RT-qPCR and n=5-6 mice/group for western blotting. *p ≤ 0.05 *vs.* WT at Basal; +p≤ *vs.* WT at 1hr post-Ex; ++p≤ *vs.* WT at 3hr post-Ex; ^$^p ≤ 0.05 *vs.* KO at 3hr post-Ex. Basal (sedentary; Basal), 1 hour (after 1h of the acute exercise; 1hr post-Ex), and 3 hours (after 3h of the acute exercise; 3hr post-Ex). a.u., arbitrary units.

The *Ppargc1-alpha* mRNA levels (**Figure 4A**) were higher for the WT 1hr post-Ex when compared to WT at Basal and 3hr post-Ex. For the KO group, the levels were higher at 1hr post-Ex when compared to 3hr post-Ex. The *Tfam* and *Nrf1* mRNA levels (**Figures 4B, C** **)** were lower for the KO group at Basal, 1hr post-Ex, and 3hr post-Ex compared to the WT group at the same time. The *Mtco1* mRNA levels (**Figure 4D**) were higher for the WT group at 3hr post-Ex when compared to Basal and 1hr post-Ex. Also, the KO at 3hr post-Ex had lower levels when compared to the WT group at the same time. Regarding the protein levels (representive membranes in **Figure 4G**), the Hsp60, TFAM, NRF1, and Mt-ND1 were not different between the groups and the times (**Figures 4E, F, H, I**). The mitochondrial complexes CV alpha subunit 2, CIII-Core protein, CIV subunit I, and CII-30kDa were not different among the groups and the times (**Figures 4J–M**). The complex I (CI) subunit NDUFB8 (**Figure 4N**) was higher for the WT group at 1hr post-Ex when compared to the IL-6 KO group at the same time.

### SR9009 treatment increase Il6ra mRNA levels

**Figure 5:**
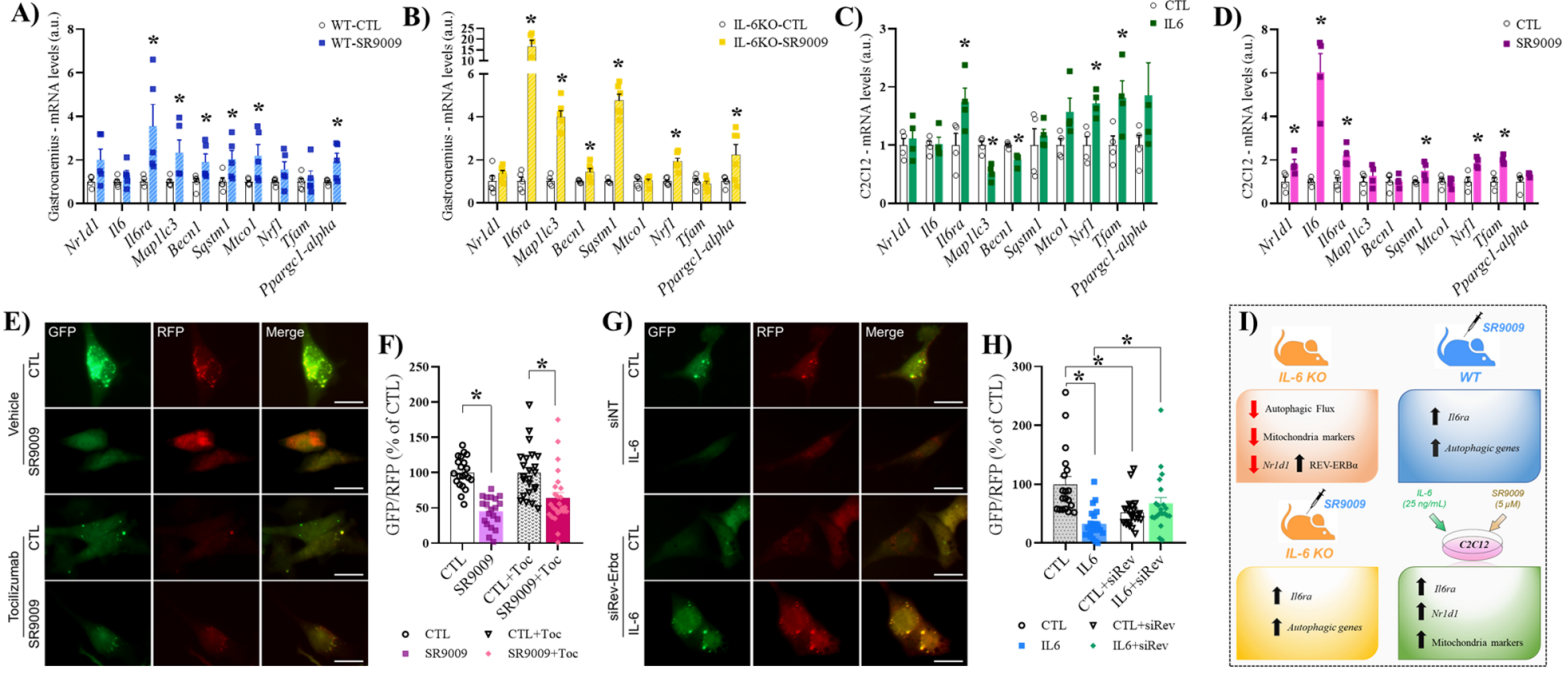
mRNA levels after IL-6 or SR9009 treatment. SR9009 treatment in WT mice (gastrocnemius) **(A)**; SR9009 treatment in IL-6 KO mice (gastrocnemius) **(B)**; IL-6 treatment in C2C12 **(C)**; SR9009 treatment in C2C12 **(D)**; Representative images of GFP/RFP in C2C12 **(E, G)**; GFP/RFP (SR9009 and Toc) **(F)**; GFP/RFP (siREV) **(H)**. Summary of key data **(I)**. Data correspond to the mean ± SEM of n=5-6 for experiments mice/group. *p ≤ 0.05 *vs.* CTL; Control (CTL), IL-6 (IL-6; received IL-6 treatment), and SR9009 (SR9009; received SR9009 treatment). a.u., arbitrary units; IL-6 KO, interleukin 6 knockout; WT, wild-type; Toc, Tocilizumab.

In the gastrocnemius of mice that received SR9009 or vehicle (**Figure 5A**), the mRNA levels of *Nr1d1*, *Il6*, *Nrf1*, and *Tfam* were not different between the groups. The mRNA levels of *Il6ra*, *Mapllc3*, *Becn1*, *Sqstm1 Mtco1*, and *Ppargc1-alpha* were higher for the SR9009 compared to the CTL group. In the gastrocnemius of IL-6 KO mice that received SR9009 or vehicle (**Figure 5B**), the mRNA levels of *Nr1d1*, *Mtco1*, and *Tfam* were not different among the groups. The mRNA levels of *Il6ra*, *Mapllc3*, *Becn1*, *Sqstm1, Nrf1*, and *Ppargc1-alpha* were higher for the SR9009 group compared to the CTL group. In the C2C12 cells treated with IL-6 or vehicle (**Figure 5C**), the mRNA levels of *Nr1d1*, *Il6*, *Sqstm1*, *Mtco1*, and *Ppargc1-alpha* were not different between the groups. The mRNA levels of *Il6ra*, *Nrf1* and *Tfam* were higher for IL-6 compared to the vehicle group (CTL) group. The mRNA levels of *Mapllc3* and *Becn1* were lower for the IL-6 compared to the CTL group. In the C2C12 cells treated with SR9009 or vehicle (**Figure 5D**), the mRNA levels of *Nr1d1*, *Il6*, *Il6ra*, *Sqstm1*, *Nrf1*, and *Tfam* were higher for the SR9009 compared to the CTL group. The mRNA levels of *Mapllc3*, *Becn1*, *Mtco1*, and *Ppargc1-alpha* were not different.

To track the autophagosome and autolysosome formation in response to IL-6 and REV-ERBα agonist, we transfected C2C12 cells with ptfLC3 plasmid and measured the autophagic flux. We observed increased autophagic flux in response to SR9009 (low GFP/RFP ratio) compared to vehicle-treated cells (**Figures 5E, F** **)**. However, in the presence of an IL-6 receptor-neutralizing antibody (Tocilizumab), the same result was observed (**Figures 5G, H** **)**. Conversely, the recombinant IL-6 treatment increased the autophagic flux compared to control cells, but this effect was lost when the cells were pre-transfected with REV-ERBα siRNA (**Figure 5F**). These findings suggest the participation of REV-ERBα protein in the IL-6-induced autophagy in C2C12 cells.

## Discussion

In the present investigation, we verified REV-ERBα, autophagy, and mitochondria pathways in the muscle of IL-6 KO mice before and after an acute exhaustive exercise, followed by treatment of IL-6 or SR9009 in C2C12 cells, as well as WT and IL-6 KO mice. In summary, the skeletal muscle of IL-6 KO mice had lower expression levels of REV-ERBα, autophagic flux, and mitochondrial genes independent of exercise status. The C2C12 cells, when treated with IL-6, did not increase the *Nr1d1* levels but upregulated some of those mitochondrial genes, reinforcing the association between IL-6 and mitochondria markers. The WT and IL-6 KO animals receiving the same agonist also upregulated *Il-6ra* and autophagic genes, demonstrating that the activation of the IL-6 receptor is independent of IL-6. When the cells were treated with the REV-ERBα agonist, IL-6 and mitochondrial genes were upregulated. The autophagic flux in C2C12 suggest the participation of REV-ERBα protein in the IL-6-induced autophagy.

Kistner et al. (Kistner et al., 2022) reported that IL-6 may behave to allocate energy. IL-6 increases energy uptake through the muscle. Consequently, in the absence of IL-6, any disruption like physical exercise should reduce performance and impair muscle recovery (1). Corroborating other data (29–33), the current investigation verified lower tolerance to the incremental load test for the IL-6 KO mice. Another possible explanation for the lower exercise tolerance is that the IL-6-STAT3 pathway may contribute to some muscular adaptations after training, such as inducing mitochondrial biogenesis and increasing mitochondrial activity. However, these effects appear specific to some skeletal muscle fiber types (1). Prolonged endurance exercise increased respiratory chain complex protein expression, which contributes to endurance performance and permits the skeletal muscle to save glycogen during exercise (4). The CI subunit NDUFB8 was upregulated for the WT group 1hr post-Ex when compared to the IL-6 KO group at the same time. The lower levels of CI for the IL-6 KO group can justify the lower exhaustion time on the treadmill, which is linked to CI activation. The CI is responsible to catalyze the transfer of two electrons from NADH to ubiquinone and is also the main site for reactive oxygen species (ROS) (5). Exercise changes the NAD+/NADH ratio, and the reduction of NADH delivers electrons to CI, justifying the increase observed in the WT group.

In response to energy stress, IL-6 releases somatic energy stored endogenously within tissue and directs it to specific tissues while negatively regulating energy expenditure exogenously [1], which does not corroborate our current calorimetric results. On the other hand, it has been previously reported (32, 34, 35) that the absence of IL-6 increased carbohydrate utilization, as shown in our values obtained by RQ (**Figure 2E**), suggesting this animal model is compensating for the poor access to somatic energy reserves (1).

The overexpression of REV-ERBα results in a decreased expression of IL-6 in macrophages (13, 14). Contrary to this data, mice lacking REV-ERBα had increased IL-6 gene expression (13, 14). These results reinforce that REV-ERBα acts as a repressor of inflammatory response in macrophages (14). The *Nr1d1* mRNA levels in the skeletal muscle were higher for the IL-6 KO group and decreased with exercise. Still, the protein levels of REV-ERBα were lower for the IL-6 KO group, independent of exercise status. Possible hypotheses for the equivocal differences between the expression of mRNA and proteins may include: 1) the acute production and degradation of regulatory proteins in response to a stimulus, as compared to housekeeping proteins that would be much longer-lived (36); 2) only 40% of the variation in protein concentrations can be explained by the mRNA quantity (36); 3) the steady-state of the protein, in other words, the protein amount may not change once translation and degradation are balanced (36, 37).

Kelly et al. (38) reported an attenuated exercise response to p-AMPK levels in the IL-6 KO mice and even in the basal state compared to control mice. AMPK is one of the primary activators of autophagy (39), and IL-6 can activate AMPK in muscle (38, 40), suggesting the lower autophagic flux index for the IL-6 KO mice occurred due to AMPK activation. Hu et al. (41) tested the effects of IL-6 on autophagic flux using the mCherryGFP-LC3B plasmid with and without chloroquine. The authors observed that IL-6 increased autophagic flux in the presence and absence of chloroquine in SW48 cells (41). We verified that mice lacking IL-6 did not have the autophagic flux modulated by acute physical exercise, reinforcing the role of this cytokine in the autophagy pathway. However, when we measured the autophagic flux in C2C12 cells to understand the participation of IL-6/REV-ERBα using the ptfLC3 plasmid, we observed increased autophagic flux in response to SR9009 (REV-ERBα agonist) compared to control cells. When SR9009 was used in the presence of Tocilizumab (IL-6 receptor inhibitor), the same result was observed. After that, we found increased autophagic flux in response to IL-6 treatment compared to control cells. On the other hand, in the presence of REV-ERBα knockdown (siRNA), this effect was lost. This data suggests the participation of REV-ERBα in IL-6-induced autophagy.

As expected, the IL-6 KO mice had lower levels of *Prkaa1* (AMPK-related gene), as verified by Kelly et al. (38). The absence of IL-6 may decrease autophagy once AMPK, an autophagy activator, is downregulated in IL-6 KO mice. However, the mRNA levels of *Becn1* were higher in IL-6 KO mice independent of exercise. In colorectal cancer, IL-6 activates autophagy by the interaction of JAK2 and Becn1 because JAK2 acts as a protein kinase that phosphorylates Becn1 at Y333 to promote autophagy initiation (41).

Endurance exercise activates PGC1α, and in the present study, we visualized an upregulation of *Pppargc1-alpha* 1 hour after the acute exercise that decreases at 3 hours post-exercise. The IL-6 KO mice showed lower *Tfam*, *Nrf1*, and *Mtco1* mRNA levels at basal and after exercise, but the mitochondrial protein contents (Hsp60, TFAM, NRF1, and Mt-ND1) were not different between groups or time. Indeed, the global deletion of IL-6 reduced the expression of mitochondrial genes concomitantly with lower levels of REV-ERBα, suggesting a mechanism of transcriptional regulation, without alterations in the mature protein content. This response may be justified by an increase in the translation process of mitochondrial proteins due to the genetic ablation of IL-6. However, the exact molecular mechanism must be further investigated. IL-6-STAT3 pathway may contribute to mitochondria biogenesis and increase mitochondria activity (1). Myotubes treated with high levels of IL-6 increase mitochondrial biogenesis, mitochondrial protein expression, and respiratory capacity (42). In the present investigation, when C2C12 myoblasts cells were treated with physiological doses of IL-6, the mRNA levels of *Tfam* and *Nrf1* were upregulated, demonstrating the association between this cytokine and mitochondrial biogenesis. Extracellular IL-6 activates the IL-6 receptor, which was also upregulated in the C2C12 cells (**Figure 5C**), increasing the phosphorylation of STAT3. This response may trigger increased mitochondrial respiration, mitochondrial biogenesis, and further STAT3 phosphorylation at tyrosine 705 (42) when stimulated by exercise.

C2C12 cells that received SR9009, an REV-ERBα agonist, had upregulation of *Tfam*, and *Nrf1* mRNA levels. In the study of Woldt et al. (11), mice with global deletion of REV-ERBα had 40% less mitochondrial DNA content in the muscle than the WT mice, indicating a controlling role of REV-ERBα on skeletal muscle mitochondrial content. These animals also showed lower mRNA content of *Tfam* and *Nrf1*, agreeing with the results verified in the present study. Woldt et al. (11) overexpressed REV-ERBα in C2C12 cells and verified increased *Pppargc1-alpha* and *Tfam* mRNA levels.

As demonstrated by Woldt et al. (11), in the absence of REV-ERBα, the mRNA levels of *Ulk1*, *Becn1*, *Atg5*, and *Bnip3* were higher in skeletal muscle. The present study did not verify an increase in the autophagic genes for the C2C12 cells treated with SR9009, but when WT mice received SR9009 for 3 days, the autophagic genes were higher than in controls. The IL-6 KO mice that received SR9009 for 3 days also upregulated the autophagic genes. Using other cell lines, such as hepatic stellate cells, the SR9009 did not change the autophagy markers (43). In mice granulosa cells, SR9009 upregulated Atg5 levels (44). The different results verified for the autophagic genes may be due to the dose of administration of SR9009, in addition to the different samples investigated.

The SR9009 treatment increased the *Il6ra* in the C2C12 cells and skeletal muscle samples, independent of IL-6 presence. Several studies (13, 14, 45) using synthetic REV-ERBα ligand, or genetic knockdown of REV-ERBα, showed a decrease in IL-6 release. However, it is essential to point out that these studies used lipopolysaccharide (LPS)-induced inflammation. Yue et al. (46) verified that SR9009 did not affect the production of inflammatory cytokines under normal physiological conditions in the hippocampus. Therefore, the state that the cells or tissue received the treatment can determine IL-6 release, mainly because IL-6 can act as an anti or pro-inflammatory cytokine.

In conclusion, global deletion of IL-6 increased *Nr1d1* mRNA levels but decreased REV-ERBα protein content in skeletal muscle. The lack of IL-6 influenced the responses of REV-ERBα, as well as autophagy and mitochondrial markers to acute physical exercise. *In vitro* and *in vivo* SR9009 treatment increased the *Il6ra* mRNA levels. These data unravel an exciting relationship between IL-6 and REVERBα, suggesting that the adaptations desired through physical exercise (increases in mitochondrial content e improvement of autophagy machinery) may be mediated by an interaction between IL-6 and REVERBα. Future investigations should evaluate the inflammatory response using REV-ERB agonists in different doses and after physical exercise. **Figure 5I** summarizes the main findings.

## Data availability statement

The original contributions presented in the study are included in the article/**Supplementary Material**. Further inquiries can be directed to the corresponding author.

## Ethics statement

The animal study was reviewed and approved by The Ethics Committee of the University of Sao Paulo (I.D. 2018.5.70.90.0).

## Author contributions

Conceptualization: AP, VM, AR, and ASRS; Data curation: AP, VM, AR, and AS; Funding acquisition: FS, GT, JP, LM, DC, ER, EF, DR, and AS; Investigation: AP, VM, AR, and RR; Methodology: AP, VM, AR, RR, GF; Supervision: AS; Visualization: AP, VM, AR, RR, GF, LA, FS, GT, JP, LM, DC, ER, EF, DR, and AS; Roles/Writing - original draft: AP, VM, and AS; Writing - review & editing: AP, VM, AR, RR, GF, LA, FS, GT, JP, LM, DC, ER, EF, DR, and AS. All authors contributed to the article and approved the submitted version.

## Funding

The present work received financial support from the São Paulo Research Foundation (FAPESP; process numbers 2017/12765-2, 2018/14818-9, 2019/11820-5, 2019/21709-4, 2020/13443-1, 2021/06291-3, 2021/08692-5, and 2021/08693-1), National Council for Scientific and Technological Development (CNPq; process number 301279/2019-5), and the Coordination for the Improvement of Higher Education Personnel (CAPES; finance code 001).

## Conflict of interest

The authors declare that the research was conducted in the absence of any commercial or financial relationships that could be construed as a potential conflict of interest.

## Publisher’s note

All claims expressed in this article are solely those of the authors and do not necessarily represent those of their affiliated organizations, or those of the publisher, the editors and the reviewers. Any product that may be evaluated in this article, or claim that may be made by its manufacturer, is not guaranteed or endorsed by the publisher.
