# Supplementary material for: IL-6 deletion decreased REV-ERBα protein and influenced autophagy and mitochondrial markers in the skeletal muscle after acute exercise
Source: Front Immunol. 2022 Oct 13;13:953272. doi: 10.3389/fimmu.2022.953272 (PMC9608639; doi:10.3389/fimmu.2022.953272)
Supplement: Supplementary file 1 [file DataSheet_1.pdf]

SUPPLEMENTARY FILE

IL-6 deletion decreased REV-ERBα protein and influenced autophagy and mitochondrial markers in the skeletal muscle after acute exercise.

Ana P. Pinto, Vitor R. Muñoz, Alisson L. da Rocha, Rafael L. Rovina, Gustavo D. Ferrari, Luciane C. Alberici, Fernando M. Simabuco, Giovana R. Teixeira, José R. Pauli, Leandro P. de Moura, Dennys E. Cintra, Eduardo R. Ropelle, Ellen C. Freitas, Donato A. Rivas, and Adelino S. R. da Silva.

Figure 6. **mRNA levels**

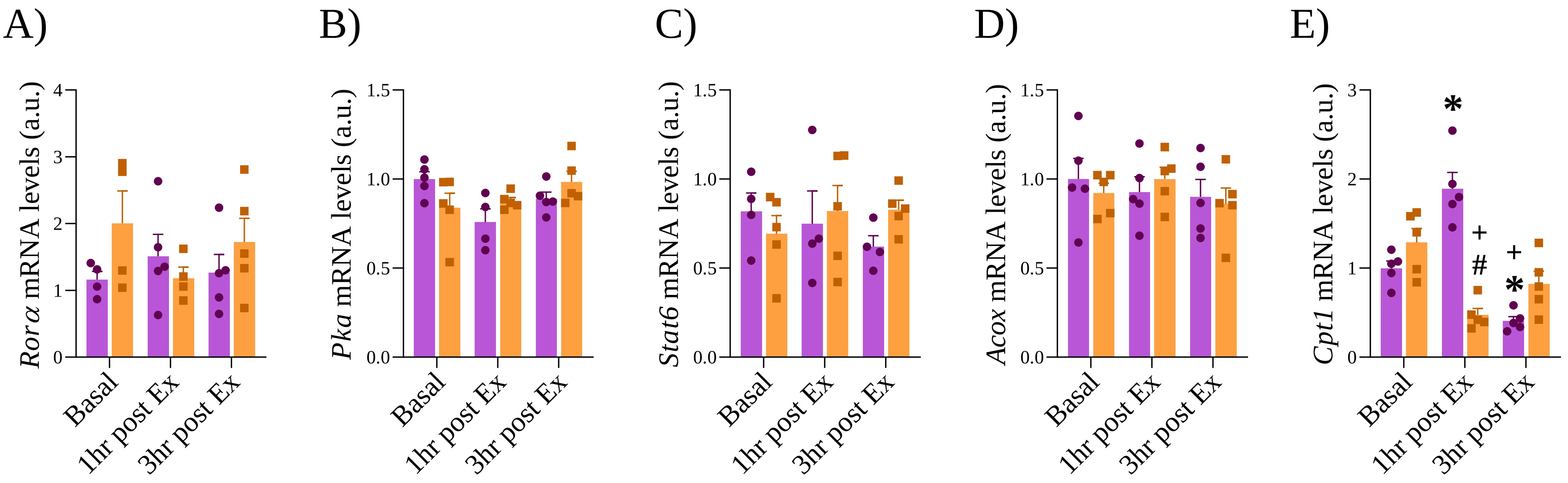

Data corresponds to the mean ± SEM of n=4-5 mice/group. *p≤0.05 *vs.* WT at Basal; #p≤0.05 *vs.* IL-6 KO at Basal; +p≤ *vs.* WT at 1hr post-Ex. Basal (sedentary; Basal), 1 hour (after 1h of the acute exercise; 1hr post-Ex), and 3 hours (after 3h of the acute exercise; 3hr post-Ex). a.u. = arbitrary units.

Figure 7. **Protein levels**

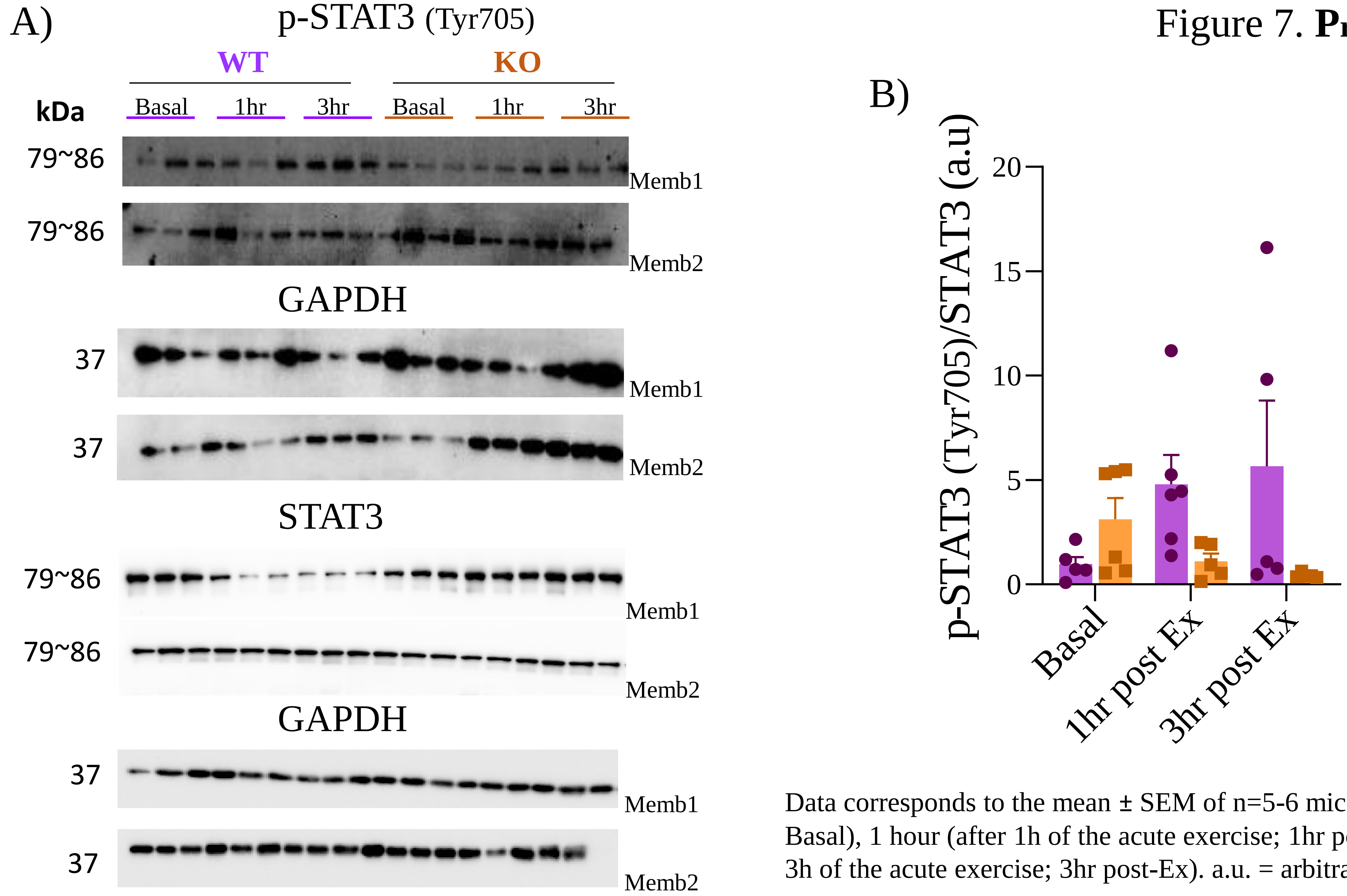

Data corresponds to the mean ± SEM of n=5-6 mice/group. Basal (sedentary; Basal), 1 hour (after 1h of the acute exercise; 1hr post-Ex), and 3 hours (after 3h of the acute exercise; 3hr post-Ex). a.u. = arbitrary units.

Figure 8. **Protein levels**

A)

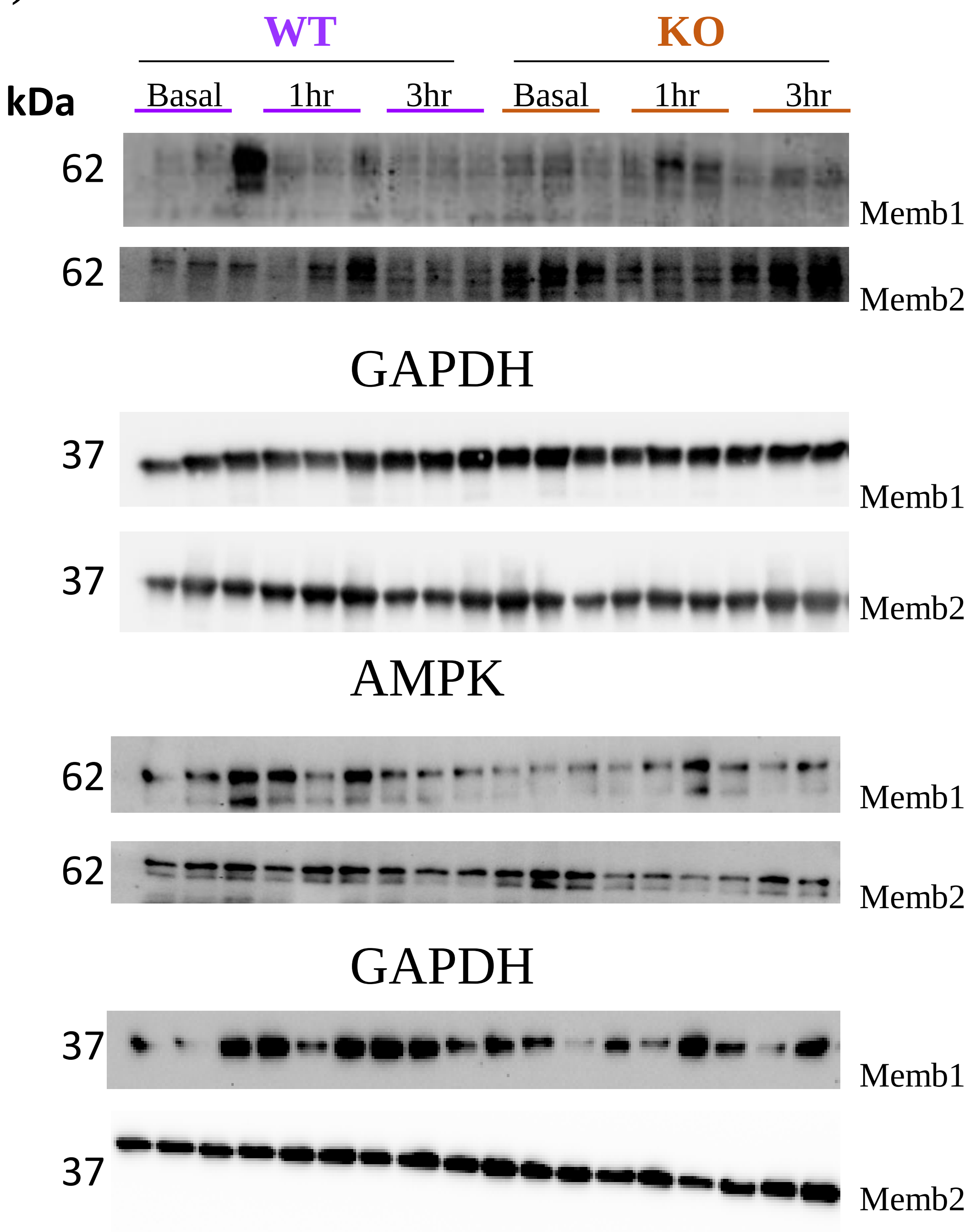

B)

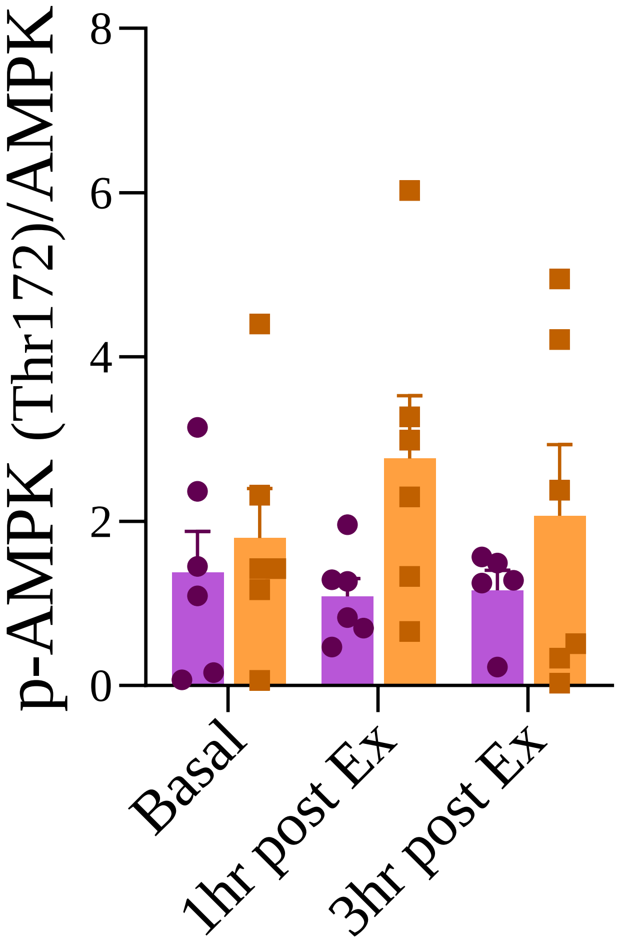

Data corresponds to the mean ± SEM of n=5-6 mice/group. Basal (sedentary; Basal), 1 hour (after 1h of the acute exercise; 1hr post-Ex), and 3 hours (after 3h of the acute exercise; 3hr post-Ex). a.u. = arbitrary units.
